# Emotional adaptation to relationship dissolution in parents and non-parents: A new conceptual model and measure

**DOI:** 10.1371/journal.pone.0239712

**Published:** 2020-10-28

**Authors:** Abigail Millings, Shannon L. Hirst, Fuschia Sirois, Catherine Houlston

**Affiliations:** 1 Centre for Behavioural Science and Applied Psychology, Sheffield Hallam University, Sheffield, United Kingdom; 2 Department of Psychology, University of Sheffield, Sheffield, United Kingdom; 3 OnePlusOne, London, United Kingdom; University of Sao Paulo Medical School, BRAZIL

## Abstract

Relationship dissolution can cause declines in emotional well-being, particularly if there are children involved. Individuals’ capacity to cope with the pragmatics of the situation, such as agreeing childcare arrangements, can be impaired. Before now, there has been no psychometric test to evaluate individuals’ emotional readiness to cope with these demands. This paper presents a model of emotional adaptation in the context of relationship dissolution and its key assumptions, and validates the Emotional Adaptation to Relationship Dissolution Assessment (EARDA). In Study 1 (Sample 1, *n* = 573 separated parents, Sample 2, *n* = 199 mix of parents and non-parents), factor analyses support the EARDA as a unidimensional scale with good reliability. In Study 2 (using Sample 1, and Sample 3, *n* = 156 separated parents) the convergent, discriminant, concurrent criterion-related, and incremental validity of the EARDA were supported by tests of association with stress, distress, attachment style, and co-parenting communication and conflict. In Study 3, the nomological network of emotional adaptation to relationship dissolution was explored in Sample 2 using cluster analysis and multi-dimensional scaling (MDS). Emotional adaptation clustered with positive traits and an outward focus, and was negatively associated with negative traits and an inward focus. Emotional adaptation was conceptually located in close proximity to active and adaptive coping, and furthest away from maladaptive coping. In Study 4 (*n* = 30 separated parents embarking on mediation), high, medium, and low emotional adaptation to relationship dissolution categories correlated highly with mediators’ professional judgement, offering triangulated face validity. Finally, in Study 5, EARDA scores were found to mediate between separation characteristics (time since break up, whether it was a shock, and who initiated the break up) and co-parenting conflict in Sample 1, supporting the proposed model. The theoretical innovation of this work is the introduction of a new construct that bridges the gap between relationship dissolution and co-parenting. Practical implications include the use of the measure proposed to triage levels of support in a family law setting.

## Introduction

Relationship dissolution is a significant stressor, which can, for some people, be traumatic, and can lead to symptoms of poor mental health [1]. When there are children involved, the nature and process of separation are different, more complex, and importantly, the implications reach far beyond the former partners and affect the well-being of the child, in the short [2, 3], medium [4], and long term [5, 6]. One of the key factors of parental separation that affects child well-being is the parents’ ability to co-parent beyond the end of the relationship. Identifying and understanding the factors that contribute to the capacity to successfully co-parent is crucial for improving the well-being of individuals and their children following separation. In a sociolegal context, Barlow and colleagues [7] have highlighted separating parents’ ‘emotional readiness’ to engage in dispute resolution as a critical factor in its success. In this paper, we elucidate the concept of emotional readiness to engage with the practicalities of separating, from a psychological perspective, specifically examining separating parents’ emotional adaptation to relationship dissolution. This new construct bridges the gap between models of relationship dissolution and theories of co-parenting. We define emotional adaptation to relationship dissolution as the degree of resolution of a person’s emotional reaction to their separation that arises from a dynamic process of coping focused on adjusting to relationship dissolution. Emotional adaptation to relationship dissolution occurs on a spectrum with low adaptation characterised by a complex mixture of predominantly negative emotions, such as anger, guilt, shame, and distress [8]. In contrast, high emotional adaptation to relationship dissolution is characterised by positive emotions. As such, emotional adaptation to relationship dissolution is inherently linked to psychological well-being.

This construct has important practical implications in the context of post-separation negotiations, where emotional adaptation is required in order to competently engage in potentially legally binding decision-making. Accurate measurement of individuals’ emotional adaptation to relationship dissolution could enable practitioners to better support clients through their separation journeys and potentially even offer intervention to help increase that adaptation. The anticipated outcome of emotional adaptation to relationship dissolution is an improvement in the individual’s capacity to engage with the pragmatic complexities of their separation, including co-parenting. Hence, emotional adaptation to relationship dissolution, while fundamental to the co-parental task and process, also has a much broader relevance to adaptation to relationship dissolution in general. Here, we outline the theoretical context and our assumptions for the construct, and in 5 studies, develop and test the Emotional Adaptation to Relationship Dissolution Assessment, employing samples of both parents and non-parents.

### Relationship dissolution

Although individuals rarely enter a relationship with the intention that it will not last, recent reports suggest that 42% of marriages in the UK [9], and 46% of marriages in the US [10], end in divorce. In the UK, half of divorces involve children under 16 years of age [11]. Research has demonstrated an association between relationship dissolution (of both married and unmarried couples) and low levels of well-being [12], poorer health [13], social isolation [14], increased risk for Major Depressive Disorder [15], increased psychological distress and a significant reduction in life satisfaction [16].

Most models that attempt to explain relationship dissolution are stage models that start with the assumption that relationship breakdown is the result of an extended process. The models proposed by Duck [17, 18] Baxter [19], Lee [20]and Knapp [21] posit that relationship dissolution occurs as a process composed of different elements that may be sequential or overlap. Knapp’s [21] model includes both the formation and dissolution of a relationship, ending with termination. Baxter’s [19] model deals only with dissolution, and similarly, ends in the decision of whether or not to terminate the relationship. Although these models include a stage beyond the termination, whereby the individual achieves a sense of resolution, they do not explain what happens next—there is no provision for psychological adaptation to the dissolution. The stage beyond termination is termed ‘resurrection’, meaning acceptance and moving on to readiness for new relationships in Duck’s [17, 18] model, and ‘transformation’, as it includes the redefining the meaning of the relationship in relation to the self in Lee’s [20] model. We propose that the construct of emotional adaptation to relationship dissolution encompasses these final stages and additionally has the potential to explain how individuals move from one stage to the next, as well as the individual differences that influence *when* they move through the stages.

### Co-parenting

We propose that an important outcome of emotional adaptation to relationship dissolution is a person’s ability to co-parent effectively. Co-parenting is defined as the pattern of attitudinal and behavioural interactions between parents that pertain to their children [22]. When parents separate, and both remain involved in their children’s lives, the co-parenting relationship necessarily endures beyond the spousal relationship, a situation which can result in any negative affect felt about the relationship and break-up being maintained, due to the enforced continued contact [23].

Co-parenting quality varies along two key dimensions—supportiveness and conflict [22]. Research suggests that children fare better following parental separation or divorce when their parents engage in supportive and cooperative co-parenting, and that the absence of this can be a risk factor for poor child outcomes, such as emotional and behavioural problems and poor academic outcomes [22]. For parents, supportive co-parenting is associated with positive post-divorce adjustment and well-being, and conflict in co-parenting is associated with poorer adjustment and poor well-being [3].

Given the implications of co-parenting, it is important to consider the antecedents of co-parenting quality. The quality of the pre-divorce relationship, the quality of the post-divorce relationship, the nature of the divorce process, custody arrangements, time since divorce, and remarriage/new family formation are key predictors of co-parenting [22]. Post-divorce relationship quality, and in turn co-parenting, may also be explained by the nature of the separation process, in terms of the level of hostility [24], and whether litigation or mediation is engaged, with litigation producing worse outcomes [25]. Time since separation/divorce is another factor that contributes to co-parenting quality. For example, most individuals recover from the negative effects of divorce and stable co-parenting routines are established within the first 2 years post-divorce [26, 27].

### Models of coping with stress

Insomuch that adaptation reflects coping with the termination of the relationship, which is assumed to be stressful particularly when there are children involved, our model of emotional adaptation to relationship dissolution is informed by models of coping and stress adaptation, and models of bereavement. According to cognitive transactional models of stress [28], stress is the result of a perceived discrepancy between the demands of a challenging, threatening or harmful event and the internal and external resources of the individual to deal with these demands. The break-up of an important relationship can be experienced as stressful insomuch that the loss of the relationship may be harmful to one’s identity [23], and threatens the viability of personal goals that involved the partner [29]. Coping theory suggests that coping is process-oriented and contextual such that the coping strategies individuals employ to manage these discrepancies can be adaptive or maladaptive, depending on the circumstances in which they are used [28].

The Vulnerability Stress Adaptation model (VSA) [30] aims to explain how within a dyad, each partner’s enduring individual vulnerabilities and strengths influence adaptive processes in response to stressful relationship events. The interaction of these components predicts relationship outcomes, such as relationship satisfaction and quality. The reciprocal effect of each aspect of the model strengthens the associations between vulnerabilities/strengths, adaptive processes, and responses to stressful events. Although it does not aim to explain what predicts, or happens in response to, relationship dissolution, it focuses on the adaptive processes that occur within relationships in response to stressors, and the effect of trait variables on state variables. Thus, the VSA offers some insight into the trait variables which predict emotional adaptation to relationship dissolution as an enduring way of responding to relationship dissolution, through adaptive processes in response to a relationship stressor.

Initially developed for death bereavement, the Dual Process Model of coping (DPM) can be applied more generally to loss experiences [31], including relationship dissolution. Following loss, orientation to everyday life experiences can be conceptualised as loss or restoration focussed. Loss orientation refers to concentrating on dealing with the loss experience itself, in this case the loss of the relationship. Restoration orientation, on the other hand, involves active coping strategies, namely focusing on dealing with issues arising from the loss. In the case of relationship dissolution where children are involved, co-parenting is an activity that would require a restoration focus because of changes in responsibility for parenting as a result of the separation. DPM assumes that individuals oscillate between a loss and restoration orientation, similar to the confrontation (loss), and avoidance (restoration) of the Transactional Model of Stress [28]. However, it also proposes that attention to additional stressors (e.g., that may be as a result of the loss) may also be necessary (e.g., looking after your joint child).

### A model of emotional adaptation to relationship dissolution

Whereas classical relationship dissolution models focus on the processes and factors that converge to result in termination of a relationship, the proposed model of emotional adaptation to relationship dissolution aims to delineate the processes involved in adaptation after the relationship dissolution, and the subsequent outcomes of this process. In short, a critical milestone in the development of emotional adaptation to relationship dissolution is when a relationship ends. We also propose that emotional adaptation to relationship dissolution is best conceptualised as an enduring but malleable way of responding to relationship dissolution, that emerges in the context of a particular relationship breakdown, and is also changeable over time. That is, the development of emotional adaptation to relationship dissolution is dependent upon the circumstances of a given relationship, but the process through which the adaptation develops is also influenced by the individual’s enduring strengths and weaknesses. This view of emotional adaptation to relationship dissolution is consistent with contemporary conceptualisations of personality that highlight the dynamic nature of individual differences and intra-personal fluctuations of feelings, thoughts, and motives in response to situational changes and changes in life circumstances [32, 33].

**Fig 1:**
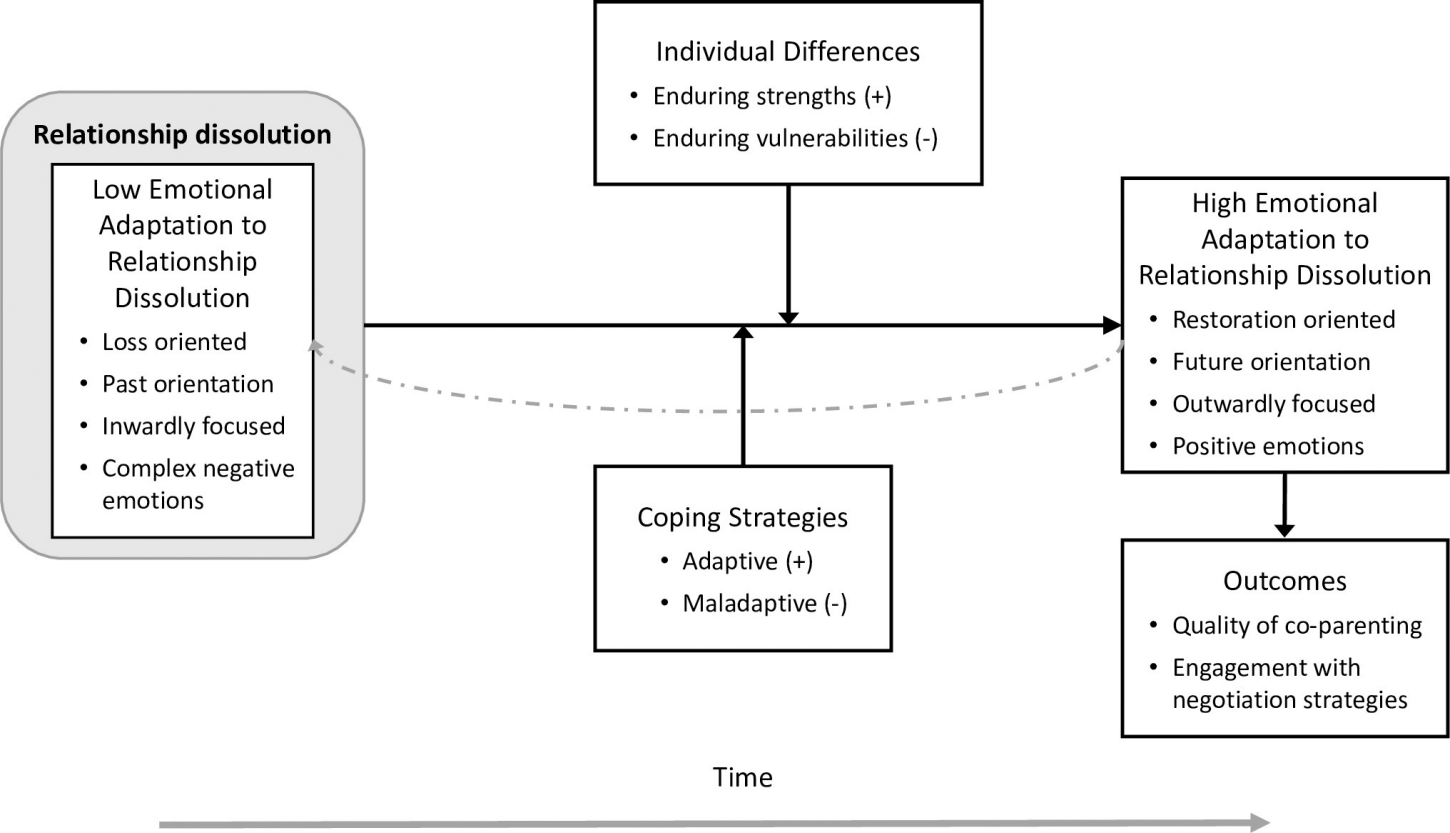
Conceptual model of emotional adaptation to relationship dissolution.

Fig 1 presents the conceptual model outlining the processes involved in the development of emotional adaptation to relationship dissolution, including moderators and potential outcomes.

Several key assumptions underpin this model and the hypotheses that it generates. In the following sections we outline these assumptions, grounding them in extant theory and research on coping and relationship dissolution.

#### Assumption 1: Relationship dissolution is a necessary contextual factor for the development of emotional adaptation to relationship dissolution (even if incomplete)

Regardless of whether or not there are children involved, emotional adaptation to relationship dissolution is assumed to develop in response to the break-up of a relationship. Given that we define emotional adaptation to relationship dissolution in terms of the individual’s reaction to a relationship break-up, this point, although somewhat obvious, is foundational for understanding the process of developing emotional adaptation to relationship dissolution. Nonetheless, it is important to note that relationship dissolution is not experienced the same way by both partners, and certainly not when one person initiated the break-up, and other received it. This invariably means that the break up may occur at different timepoints for each individual, and former partners will have relatively different levels of emotional adaptation to relationship dissolution at the point of relationship dissolution. The significance and investment in the relationship [16], whether children are involved [34], attachment to the ex-partner [35], the extent to which the break-up was a shock, and accordingly whether one was the initiator or not [36], are important considerations when understanding a person’s level of emotional adaptation to relationship dissolution. It follows, then, that each break-up a person experiences may produce a different starting point for emotional adaptation to relationship dissolution, depending on the non-exhaustive contextual factors outlined above. However, it is also likely that a person’s trait-like enduring strengths and vulnerabilities will produce a level of intra-personal correlation with emotional adaptation following different break-up experiences.

#### Assumption 2: Individuals start from a position of low rather than high emotional adaptation to relationship dissolution following the dissolution of a relationship

This key assumption has its basis in research that demonstrates that relationship dissolution is generally experienced as an unpleasant and challenging life event that evokes psychological distress and creates vulnerability for poor well-being [12, 16]. That is not to say that the dissolution of some relationships, such as those involving intimate partner violence, or chronic infidelity i.e., those relationships involving ‘major relationship transgressions', [37, 38] can result in positive affective responses. However, research suggests that even these separations can be traumatic [39], and we propose that emotional adaptation to relationship dissolution remains a relevant construct within this context. Consistent with models of stress and coping [28], emotional adaptation to relationship dissolution is therefore assumed to be relatively low at the point of the relationship breakup, as the individual initially experiences the threatening and harmful aspects of this stressful life event. Low emotional adaptation can therefore be viewed as the starting point experienced following the break-up. The experience of the negative emotions associated with low emotional adaptation are considered to be antithetical to resolving one’s emotional reactions to the break-up and engaging with the pragmatic complexities of the relationship dissolution. However, as noted in Assumption 1 there can be variability in the levels of emotional adaptation that people experience after a break-up. For example, those whose relationship was toxic or abusive may be in higher state of emotional adaptation at the point of dissolution, especially if they are the initiator of the break-up.

In addition to being characterised by predominantly negative emotions, low emotional adaptation to relationship dissolution involves a set of cognitive orientations akin to those experienced during bereavement. Similar to the Dual Process Model of coping (DPM; [31]) we propose that after a relationship break-up individuals experience a loss-oriented mind-set that places focus predominantly on the loss of the relationship. Expanding on this view, we further posit that a loss-oriented view is predominantly past-oriented, as the individual reflects on past positive and/or negative experiences from the relationship that are now lost. This reflective stance can also be described as inward focused, as the individual is more focused on the self rather than others, and is concerned more with memories than future planning. This set of cognitive orientations may further contribute to the complex set of negative emotions experienced when emotional adaptation to relationship dissolution is low, by focusing attention to loss and feelings of regret over a relationship that has come to an end.

#### Assumption 3: The movement from low to high emotional adaptation to relationship dissolution involves shifts from (i) being loss-oriented to restoration oriented, (ii) a past orientation to a future orientation, (iii) an inward to outward focus, and (iv) a balance favouring negative emotions towards one favouring positive emotions

As noted in Assumption 2, low emotional adaptation includes a particular cognitive orientation accompanied by a complex set of negative emotions. As depicted in Fig 1, developing emotional adaptation involves a process of moving towards being emotionally ready to deal with the pragmatic aspects of the relationship dissolution, which involves in part a shift in cognitive orientation and emotional valence. Accordingly, we propose that reaching high emotional adaptation entails a process of transitioning to a restoration focus from a loss focus, in much the same way that the DPM [31] outlines adjustment to bereavement. In the context of relationship dissolution, a restoration focus involves active coping and dealing with the aftermath of the break-up. We also propose that other cognitive orientations that are consistent with a restoration focus characterise high emotional adaptation, including being more future than past oriented, and more outwardly (world, others) than inwardly (self) focused. As with low emotional adaptation, these particular cognitive orientations may influence emotional states in a reciprocal and dynamic manner. For example, future orientation can foster positive emotional states and vice versa [40]. Lastly, high emotional adaptation to relationship dissolution does not assume the absence of all complex negative emotions experienced when emotional adaptation is low. Indeed, it would be unrealistic to expect that all residual negative emotions from the break-up would dissipate easily or quickly. Rather, high emotional adaptation to relationship dissolution is posited to involve a shift towards predominantly positive as opposed to negative emotions about the relationship loss being experienced. The positive emotions that characterise high emotional adaptation to relationship dissolution may also be less complex than those experienced when in low emotional adaptation, and focus on feelings of hope and perhaps relief that the bulk of the difficulties lie behind rather than ahead.

#### Assumption 4: Changes in emotional adaptation to relationship dissolution occur over time, and are facilitated by the use of adaptive coping strategies

Having described the beginning and end points of emotional adaptation to relationship dissolution it is also important to detail the factors which can facilitate or deter the process of achieving emotional adaptation. Reaching high emotional adaptation to relationship dissolution implies that adaptive coping strategies have been identified and utilised to minimise the stress associated with the break-up and facilitate adjustment. Such a process would necessarily occur over time, with greater emotional adaptation arising in part from the use of adaptive coping strategies, including emotion-focused strategies, acceptance of the break-up, and emotional or social support-seeking strategies, which focus on reducing the negative emotional experience of the break-up. However, it is also likely that the use of less adaptive emotion-focused coping strategies would deter the development of emotional adaptation to relationship dissolution. For example, avoidant strategies such as denial and disengaging from coping efforts may provide relief from distressing feelings in the short term, but do little to reduce the stress from the stressor (break-up) in the long–term [41]. In addition, self-defeating strategies such as self-blame, which are known to contribute to poor adjustment in the context of ongoing stressors [42] would also be expected to interfere with the transition from low to high emotional adaptation to relationship dissolution. Having reduced the initial stress and concurrent negative emotions from the dissolution of the relationship, the individual would have an increased capacity to engage with more problem-focused coping efforts aimed at managing the practical and potentially complex aspects of the break-up.

#### Assumption 5: Individual differences in enduring strengths and vulnerabilities facilitate or hinder (respectively) the transition from low to high emotional adaptation to relationship dissolution

Aside from coping strategies, moving from low to high emotional adaptation is assumed to be dependent on the set of particular strengths and vulnerabilities that the individual possesses and how they influence adaptation to the relationship break-up. This notion is similar to that proposed by the Vulnerability Stress Adaptation (VSA) model [30], whereby such individual differences can strengthen or weaken adaptive processes to the stressful relationship event (in this case break-up). Within the model of emotional adaptation to relationship dissolution, we conceptualise these strengths and weaknesses as moderating the speed of transition from low to high adaptation. For example, traits and qualities associated with psychological flexibility, trust in others, and positive views of the self would be expected to facilitate movement from low to high emotional adaptation by providing the internal resources necessary to navigate the complex emotions and coping processes involved [41]. In contrast, traits and qualities associated with avoidant tendencies, psychological rigidity, negative self-views [43], and perfectionism [44], are expected to hinder the process of developing emotional adaptation. This is due to them making it difficult for the individual to move beyond a preoccupation with the negative thoughts and emotions that arise from the experience of relationship dissolution.

#### Assumption 6: The transition from low to high emotional adaptation to relationship dissolution may follow a dynamic, non-linear pathway involving cycling back to lower levels of adaptation

Implicit within our model of emotional adaptation to relationship dissolution is the idea that the journey from low to high emotional adaptation does not necessarily occur in a linear manner, but may involve cycling back to states of lower emotional adaptation as part of a natural cycle of adjustment to the stressful event. This idea is predicated on the dynamic process of adjustment to loss described in the DMP. [31]. This model envisions coping with loss (for our purposes, relationship break-up) as an oscillating process between loss and restoration orientation. As noted in Fig 1, the dashed arrow accounts for the possibility of returning to a state of lower emotional adaptation, which may occur in the presence of additional environmental stressors, or simply as part of a natural cycle of coping. To the extent that returning to lower states of emotional adaptation are temporary and may facilitate the establishment of a more stable emotional adaptation via emotional processing, then such backswings can be viewed as adaptive.

#### Outcomes of emotional adaptation to relationship dissolution

Although not specifically an assumption, our definition of emotional adaptation indicates that an improvement in the individual’s capacity to engage with the pragmatic complexities of their separation, including co-parenting, is an expected outcome of developing greater emotional adaptation to relationship dissolution. We envision that there are several beneficial outcomes that might follow from having high emotional adaptation, and these can be generally viewed as falling under two broad categories: 1) good quality of co-parenting (when children are involved), and 2) better engagement with negotiation strategies. These ideas are based on socio-legal research observations that conflict resolution strategies such as mediation tend to fail when clients are not emotionally ready to engage with them [7].

Having a high degree of emotional adaptation to relationship dissolution involves turning from an inward, self, past, and loss focus, to a focus more on the outward realities of the relationship dissolution, being restoration-focused, future-oriented, and child-focused; an orientation that should be conducive to working through the practicalities of arranging and maintaining a good standard of co-parenting quality, including making equitable custody arrangements. The shift from complex negative emotions to more positive emotions are beneficial for co-parenting quality, and because negative emotions can contribute to conflict and subsequent poor child outcomes [22, 45].

High emotional adaptation to relationship dissolution may also be conducive for engaging with, rather than avoiding, adaptive negotiation strategies, regardless of whether or not there are children involved in the relationship dissolution. Law researchers have theorised that one reason for mediation failing is likely to be low emotional readiness (in one or both parties) to engage with the process [7]. Research into this idea is lacking, at least in part because until now there has been no clear conceptualisation of separating parents’ emotional readiness to engage in such practicalities, or how to measure it. We propose our new psychological construct of emotional adaptation to relationship dissolution, and scale with which to measure it, fills this gap.

### The current research

We have presented our model of emotional adaptation to relationship dissolution. Next, we describe the development of a scale designed to measure this novel construct. In Study 1, we created and test the newly developed Emotional Adaptation to Relationship Dissolution Assessment (EARDA). In Study 2, we examined the construct, criterion, and incremental validity of the EARDA. In Study 3, we further examined its construct validity by investigating the nomological network of the emotional adaptation to relationship dissolution construct in a mixed parent and non-parent sample. In Study 4, we demonstrate the use of the EARDA in an applied setting and assess its concordance with professional mediators’ opinions. Finally, in Study 5 we examine the extent to which EARDA scores can account for differences in key outcomes related to co-parenting after relationship-dissolution, as proposed by our model.

## Study 1

An important and necessary first step in the development of a scale to measure a new construct is to create and test a set of items that are designed to reflect the theoretical breadth of the construct [46]. From the perspective of the deductive method of item generation, items generated “top down” from theory should be closely aligned with the theoretical description of the construct [47], while at the same time avoiding items that provide repeated coverage of the same ideas to create “bloated specifics” [48]. In contrast, the inductive method relies on “bottom up” generation of items based on the responses of individuals with insights and experience relevant for the construct being measured [47]. A recommended best practice is to combine the deductive and inductive approaches [49]. After generating an item pool, items proposed for the new scale are then subjected to tests to assess scale dimensionality [50]. Accordingly, in Study 1, we used a combined deductive and inductive approach to develop and screen items, and then assess the underlying factor structure of our new measure, the Emotional Adaptation to Relationship Dissolution Assessment (EARDA).

### Study 1 method

#### Participants and procedures

Participants for Sample 1 were recruited by Qualtrics, which secures research participation panels using third party partners, offering a range of incentives, such as air miles, redeemable points, gift cards, sweepstake entrance, vouchers, and cash. Participants for Sample 1 were 573 parents who had separated from the other parent of their child(ren) at some point in the last 6 years; 176 (31%) were male, 397 (69%) were female. Dyads were not recruited. Participants’ ages ranged from 20 to 63 (*M* = 37.96, *SD* = 8.82). Men (*M* age = 41.32, *SD* = 8.71) were significantly older than women (*M* age = 36.47, *SD* = 8.46), *t*(571) = 6.27, *p* < .0001.

For Sample 2, participants (*N* = 199; 146 = female; 49 = male; 2 = gender fluid; 1 = transgender male) were recruited using snowball sampling via an advertisement to the University of Sheffield’s staff and student research volunteer list, and via advertisements shared on the research team’s social media (i.e., Twitter, Facebook) and other online recruitment platforms (i.e., Reddit, The Student Room, Psychology Research on the Net). Participants’ ages ranges from 18 to 70 (M = 28.80, SD = 9.89), there were no significant differences in age between male and female participants, *t(193) =* .*30*, *p =* .*76*. The majority of participants did not have children (*n* = 160). Study requirements asked that participants be over 18 and have experienced the break-up of a significant romantic relationship. Participants were offered the opportunity to enter into a prize draw for a £30 Amazon voucher to thank them for their participation.

Ethical approval for the study was received from The University of Sheffield Ethics Committee (Psychology) prior to data collection. Participants provided informed consent before completing a survey consisting of demographic items and self-report scales. We also collected data on a number of self-report scales for each sample for the purposes of validity testing. Details of these scales and the data obtained from them are reported in Study 2 and Study 4.

#### Measures

Participants took part through the Qualtrics platform and were asked to complete a range of self-report measures in an online questionnaire format.

*Demographics*. Participants were asked to report their age, gender, the nature of the relationship they had had with their child(ren)’s other parent ‘(Sample 1) or their ex-partner (Sample 2), the relationship length in years, and the amount of time (in years and months) since the separation occurred (defined as the time when both parties became explicitly aware of the decision). Participants were also asked to report who initiated the separation (me, my ex-partner, it was mutual, and I’d rather not say) and whether it was a shock (yes, I could see it coming, and not sure). Participants in Sample 1 reported the number of children they had, and where the children lived. Participants in Sample 2 reported on the significance of the relationship from 0% (Not at all significant) to 100% (Very significant).

*Emotional adaptation to relationship dissolution*. We used a combined theory-driven (deductive) and applied (inductive) approach to create items to measure our new construct of emotional adaptation to relationship dissolution. Items were designed to tap the emotional state of parents’ post-separation with regard to planning and making childcare arrangements with their ex-partner. However, items were also carefully worded so as to be more broadly applicable to any individuals who had experienced a relationship break-up. Items were created through literature reviewing and consultation with family psychotherapists and trained mediators with experience in family law. Twelve items were created, 10 of which were retained after roundtable discussions to screen for content validity. Two of the items did not adequately map onto the emotional adaptation to relationship dissolution construct and were therefore removed [51].

Participants were asked ‘From 0% to 100%, How much does each of these statements describe how you are feeling about your break-up now?’ and rated the 10 items on visual analogue scales from 0% (‘Does not describe my feelings at all’) to 100% (‘Describes my feelings exactly’). Continuous VAS scales were selected rather than 1–7 point Likert scales as they can be superior for capturing subjective experiences [52], increase variance [53], and are no more time-consuming than Likert scales to score when implemented in an online environment. Negative valence items were reverse coded, and mean scores calculated, with higher scores indicating greater emotional adaptation to relationship dissolution.

### Analytic strategy

The strategy adopted for the factor analysis of the EARDA follows the inductive approach suggested by [50]. To assess the underlying factor structure of the scale, we conducted factor analyses using an unweighted least squares extraction. An unweighted least squares approach therefore minimises the influence of the error variance in the selection of factors, and in this respect produces more accurate estimates of the factor loadings than a principal components analysis [54]. As the factor structure was unknown, we used an oblimin rotation to allow potential factors to be correlated [55]. In addition, we conducted a check of the simple factor structure using [48] scree test. After generating a scree plot of the eigenvalues that correspond to each factor, visual inspection was used to determine the number of meaningful factors within each dimension subscale. Cattell’s [48] guidelines suggest retaining factors that are at or above the visual elbow” of the plot, and rejecting those factors that lie below it. A useful heuristic for identifying the “elbow” is to observe the point at which the angle on the line is most severe. Although this test is considered highly subjective, it avoids the overestimation of factors that other tests promote, such as Kaiser’s eigenvalue rule [56]. (Kaiser’s eigenvalue rule involves retaining factors based on the magnitude of the factors’ eigenvalues, and thus retains factors that explain more variance than the average amount explained by any one of the items [50]. Usually the number of eigenvalues greater than one are taken to indicate the number of factors underlying the model).

DeVellis [57] suggests that a replication of the scales’ reliability and factor structure with an independent sample is useful for demonstrating factor stability. Accordingly, we randomly split the sample into two smaller subsamples: Sample 1a (*n* = 309) and Sample 1b (*n* = 264) to cross-validate the factor structure. We then further validated the factor structure in Sample 2 (*n* = 193), a sample of parents and non-parents.

### Study 1 results and discussion

#### Descriptive statistics

For Sample 1, the length of time that participants were in a relationship with their child(ren)’s other parent ranged from 0 to 32 years (*Mdn* = 9.00, *IQR* = 9), other relationship relevant descriptive statistics are described in Table 1. The living situation of participants’ children differed as a function of gender (χ^2^(3) = 290.36, *p* < .001). Inspection of the standardized residuals showed that men were less likely than would be expected by chance to report that their child(ren) lived mainly with them (*z* = -7.5), more likely (than chance) to report that their child(ren) lived with their ex-partner (*z* = 11.2), and more likely (than chance) to report that their child(ren) lived equally between themselves and their ex-partner (*z* = 4.4). Whereas, women reported the opposite pattern of results. The living situation of participants’ children also differed as a function of who initiated the break up (χ^2^(3) = 62.91, *p* < .001). Men were less likely than would be expected by chance to report that they had initiated the separation (*z* = -4.4), more likely (than chance) to report that their ex-partner initiated the separation (*z* = 3.2), and more likely (than chance) to report that it was mutual (*z* = 3.4). Again, women reported the opposite pattern of results.

**Table 1:** Descriptive statistics and frequencies for Sample 1, 1a, 1b (from Study 1).

Variable		Sample 1	Sample 1a	Sample 1b	Sample 2
		*n* = 573	*n* = 264	*n* = 309	*n* = 199
Mean Age		37.96 (8.82)	38.17 (*SD* 8.66)	37.78 (*SD* 8.97)	28.80 (*SD* 9.89)
Gender					
	Male	176 (31%)	89 (33.7%)	87 (28.2%)	50 (25.1%)
	Female	397 (69%)	175 (66.3%)	222 (71.8%)	146 (73.4%)
	Other	0 (0%0)	0 (0%0)	0 (0%0)	3 (1.5%)
Years since separation		2.91 (1.53)	2.85 (*SD* 1.54)	2.96 (*SD* 1.51)	3.60 (SD 3.84)
Relationship with ex					
	Married	274 (48%)	131 (49.6%)	143 (46.3%)	15 (7.5%)
	Cohabiting	265 (46%)	118 (44.7%)	147 (47.6%)	56 (28.1%)
	Serious non-cohabiting*	-	-	-	124 (62.3%)
	Other	34 (6%)	15 (5.7%)	19 (6.1%)	4 (2%)
Significance of relationship*		-	-	-	80.64 (*SD* 19.85)
Initiated separation					
	Self	281 (49%)	120 (45.5%)	161 (52.1%)	87 (43.7%)
	Ex-partner	151 (26.4%)	77 (29.2%)	74 (23.9%)	85 (42.7%)
	Mutual	134 (23.4%)	65 (24.6%)	69 (22.3%)	21 (10.6%)
	Rather not say	7 (1.2%)	2 (.8%)	5 (1.6%)	1 (.5%)
Was separation a shock?					
	Yes it was a shock	149 (26%)	73 (27.7%)	76 (24.6%)	71 (35.7%)
	I could see it coming	402 (70.2%)	181 (68.6%)	221 (71.5%)	115 (57.8%)
	Not sure	22 (3.8%)	10 (3.8%)	12 (3.9%)	9 (4.5%)
Where do/does child/children mostly live?*					
	Mainly with me	384 (67%)	175 (66.3%)	209 (67.6%)	-
	Mainly with partner	112 (19.5%)	54 (20.5)	58 (18.8%)	-
	Shared roughly equally	55 (9.6%)	28 (10.6%)	27 (8.7%)	-
	Other	22 (3.8%)	7 (2.7)	15 (4.9%)	-
Mean Total ER score		56.04 (*SD* 21.92)	56.04 (*SD* 21.94)	56.04 (*SD* 21.94)	64.94 (*SD* 21.02)

* ‘Serious non-cohabiting’ was not a response option included in Sample 1, and ‘Where do/does child/children mostly live?’ was not asked of Sample 2.

The descriptive statistics for the total Sample 1 and each randomly selected subsample are presented in Table 1.

For Sample 2, relationship length ranged from 1 month to 21 years (*M* = 1.63, *SD* = 3.51) and significance of relationship was overall high (*M* = 81.85, *SD* = 17.65). Other relationship relevant descriptive statistics are described in Table 1. Chi squares indicated that neither the frequencies for break-up initiation category, χ^2^(6) = 6.13, *p* = .41 or shock category, χ^2^(4) = 2.46, *p* = .65, differed by gender.

*Factor structure of the emotional adjustment to relationship dissolution assessment*. Kaiser-Meyer-Olkin (KMO) tests of sampling adequacy indicates the amount of variance in the variables that could be attributed to underlying factors. KMO ranges from 0–1 and testing for all three samples indicated that they were of sufficient size for testing factor structure: Sample 1a the KMO = .84, Sample 1b the KMO = .83, and Sample 2 the KMO = 78 [58]. In each of the three samples, the scree plot indicated one factor, with a clear “elbow” showing just below the first factor. The lack of a clear multidimensional factor structure was further demonstrated by inconsistencies in the number and quality of factors extracted across the samples. In Samples 1a and 1b, the rotated factor matrix suggested a two-item, second factor, consisting of the items related to anger and resentment. In Sample 3 the rotated factor matrix proposed 3 factors with five items loading onto the first factor (relief, hopefulness, frustration, anxiousness, loss), three items loading onto one factor (guilt, shame, and perceived failure), and two items loading onto the third factor (anger, resentment). However, a minimum of three items per factor is the general recommendation when identifying factors in a factor analysis [59].

Given these inconsistencies and the scree plots, the factor analysis was rerun for each sample using a principal components analysis and specifying one factor. The results of the factor analysis are presented in Table 2. In Samples 1b and 2 all items loaded above .30, a cut off considered the minimum for an item to be included on a factor [50]. In Sample 1a, two items (resentful, angry) loaded below .30. However, factor loadings are only one source of information when deciding the factor structure of a new scale. The meaningfulness of the items with respect to the construct being measured is also an important consideration when making decisions about item retention and factor structure [50]. Low emotional adaptation to relationship dissolution is posited to include a set of complex negative emotions. Anger and resentment are key emotions within this set that are particularly meaningful for individuals who did not instigate the break-up. In light of these important conceptual considerations, all 10 items were retained for the new EARDA scale and emotional adaptation to relationship dissolution was deemed to be a unidimensional construct.

**Table 2:** Emotional adaptation to relationship dissolution items and their factor loadings across three samples.

	Sub-sample 1a	Sub-sample 1b	Sample 2
	*N* = 309	*N* = 264	*N* = 199
Items			
9. I feel a failure that my relationship broke down. R	.852	.826	.757
5. I feel I can’t get over what I have lost. R	.806	.797	.824
1. I feel ashamed I couldn’t keep the relationship together. R	.792	.751	.627
8. I feel frustrated by my situation. R	.773	.803	.822
4. I feel anxious about what will happen next. R	.740	.757	.794
10. I feel hopeful about the future.	-.651	-.652	-.522
3. I feel relieved the relationship is over.	-.632	-.605	-.553
2. I feel guilty I broke up the relationship. R	.592	.420	.319
7. I feel resentful towards my ex-partner. R	.281	.447	.370
6. I feel angry at my ex-partner. R	.241	.426	.450

R = reverse scored items.

We next calculated the Cronbach’s alpha to determine whether the EARDA scale was internally consistent as a unidimensional scale (i.e., that the items included in the scale measure the same underlying construct). As the overall aim of the development of the EARDA was for it to be used in applied settings, a minimum alpha level of α = .80 was deemed acceptable [56]. The coefficient alpha was above this level in Sample 1a (α = .85), 1b (α = .84), and 2 (α = .82) suggesting a good internal reliability of the EARDA in all samples. Furthermore, in all samples, EARDA correlated with time since break up (Sample 1a, *r* = .20, *p* = .001; Sample 1b, *r* = .21, *p* < .001, Sample 2, *r* = .38, *p* < .001).

The results from three factor analyses indicate that the EARDA is a unidimensional scale with good internal consistency. Having established the factor structure of the EARDA we next set out to test various aspects of the construct validity of the EARDA in Studies 2 and 3.

## Study 2

The aim of Study 2 was to provide an initial assessment of the construct validity, criterion-related validity, and incremental validity of the newly created EARDA using a new dataset as well as data from Sample 1 collected for Study 1. Two key aspects of construct validity are convergent and discriminant validity, which establish what a test measures and what it does not measure, respectively [60]. This is often accomplished by comparing the new scales with other general personality measures and observing the patterns of correlations [61]. Accordingly, related constructs should show higher correlations with the scales (*r* ≥ .30), and unrelated or distinct constructs should show low associations (*r* < .20) [62].

To assess convergent validity, we examined the correlations between EARDA score and measures of psychological distress and stress. Our expectation was that the EARDA would be negatively associated with each, due to the known links between relationship dissolution and poor well-being [12] and increased psychological distress [16]. This prediction is consistent with our proposition that relationship dissolution is a stressful event and that low emotional adaptation is expected when experiencing this stressful event.

We also examined correlations with attachment style. There is ample evidence that insecure attachment orientations are associated with maladaptive relationship behaviours [63] and suboptimal parenting styles [64]. Such behaviours can affect the ability to co-parent successfully. We examined attachment anxiety for convergent validity, and attachment avoidance for discriminant validity (below). Attachment anxiety is a hyperactivating affect regulation style featuring rumination about relationships, heightened negative affect, and negative self-views [65]. Attachment anxiety is associated with low trust [66] and poor emotion regulation [67]. Attachment avoidance is associated with distancing strategies, which can manifest as low empathy [68] and relationship destructive behaviours [69]. Given these patterns of relationship dynamics, both attachment anxiety and avoidance can impact one’s ability to successfully co-parent in the face of a relationship breakdown.

We expected to see a moderate, negative correlation between emotional adaptation to relationship dissolution and attachment anxiety. Our reasoning was that emotional adaptation represents the reduction of negative emotional reactions to the break-up and an increase in feelings of hope and optimism, both in opposition to attachment anxiety. To assess discriminant validity, we examined the association of emotional adaptation to relationship dissolution with attachment avoidance, which is a deactivating affect regulation strategy, featuring suppression of relationship-related cognitive and affective responses [65]. Given that emotional adaptation to relationship dissolution is proposed to involve emotional reactions to relationship dissolution, rather than suppression of these, we expected to see no, or a very small negative correlation with attachment avoidance.

One aspect of criterion-related validity is concurrent validity, or the extent to which current scores on a measure estimate an individual’s present criterion score [61]. This form of criterion-related validity is characterized as the practical validity of a test in a specific situation [70]. Given the proposed implications of emotional adaptation to relationship dissolution for outcomes such as the quality of co-parenting, we examined the associations of emotional adaptation with co-parenting items to assess validity. Lastly, we supplement these traditional tests of psychometric validity with a test of the scale’s incremental validity. Incremental validity is “the degree to which a measure explains or predicts a phenomenon of interest relative to other measures” [71]. In this case, the phenomenon of interest was capacity to co-parent effectively, operationalised as the ability to manage emotions and communicate calmly with the ex-partner, and co-parenting conflict. In addition to Sample 1 from Study 1, a new Sample 3 was collected to supplement the variables available in Sample 1 to assess the various forms of validity described above.

### Study 2 method

#### Participants and procedures

*Participants for Sample 1 were those described in Study 1*. *Participants for Sample 3* were *N* = 156 parents who were separated from the other parent of their child(ren). Time since separation ranged from .08 years to 12.92 years (*M* = 3.91, SD = 3.49). Sixty-one participants were male, 94 were female, and 1 preferred not to say. Participants’ ages ranged from 20 to 58 (*M* = 41.46, *SD* = 8.33). Men and women did not differ in age, *t*(153) = -.09, *p* = .93. Participants were recruited via an advertisement to a university staff research volunteers list, and an advertisement on a relevant charity’s webpages. Participants had the option to be entered into a prize draw for Amazon vouchers (2 x £50) to thank them for their participation. Ethical approval for the study was received from The University of Sheffield Ethics Committee (Psychology) prior to data collection, and all participants indicated their consent to participate. It should be noted that these two samples completed different measures (see Table 3).

**Table 3:** Descriptive statistics for Study 2.

Sample 1	M (SD)	α
EARDA	56.04 (21.92)	.85
Conflict	10.84 (4.41)	.89
Co-parenting experience	14.48 (7.01)	.96
Attachment anxiety	4.23 (1.57)	.86
Attachment avoidance	3.88 (1.36)	.90
Sample 3	M (SD)	α
EARDA	59.71 (20.45)	.92
Psychological distress (PHQ4)	4.25 (3.62)	.72
Stress (PSS)	7.24 (3.32)	.82

#### Measures

In addition to the new EARDA scale, participants for Sample 1 completed the following measures.

*Co-parenting quality*. Co-parenting quality was measured using two different measures:

***Quality of Co-parental Communication*, *Conflict sub-scale*** [72]. Part of a longer measure of co-parenting communication, this sub-scale comprises 4 items, rated from 1 (never) to 5 (always). Mean scores are calculated, and higher scores indicate greater levels of conflict.

***Experiences of Co-parenting Scale*** [73]. The Experiences of Co-parenting Scale (ECS) measures respondents’ satisfaction with different aspects of their co-parental relationships. The four highest loading items from this scale were used [73]. The phrase ‘Co-parenting with my ex-partner is….’ was completed with ratings from 1–7, with a middle anchor of neutral, positive responses to the right and negative responses to the left. Responses indicated how good/bad, successful/unsuccessful, easy/hard, and relaxed/tense it is perceived to be. Total scores are created by calculating the sum of the means of each subscale. Higher scores indicate more positive parenting experiences. Reliability of the ECS is good, ∝ = .95 [73].

*Attachment style*. Attachment style was measured using the Experiences in Close Relationships– 12 (ECR-12) [74]. This measure is a shortened version of the Experiences in Close Relationships scale [75], the most widely used adult attachment self-report scale. The ECR-12 has 2, 6 item scales, one measuring attachment avoidance (‘I don’t feel comfortable opening up to others.’) and one measuring attachment anxiety (‘I worry a fair amount about losing my relationships.’). Each item is rated on a 1 (*strongly disagree*) to 7 (*strongly agree*) point scale. Mean scores are calculated for each scale. Items were presented in a randomised order. Due to a questionnaire error, the one item from the anxiety scale was not displayed, and another item from the same scale was instead displayed twice. Mean scores were calculated as usual, using both responses to the duplicated item. Reliability for the ECR-12 is good for both the anxiety (∝ = .87) and avoidance (∝ = .79) subscales [74].

Participants for Sample 3 completed a range of self-report measures in an online questionnaire format.

*Demographics*. Participants completed the same demographic questions as in Study 1, with the exception of nature of relationship, who initiated the separation, and whether the separation was a shock or not, which were not collected in this study. Due to a technical error in the questionnaire, data on children’s living arrangements was missing for 68 participants.

*Emotional adaptation to relationship dissolution*. The 10-item EARDA developed in Study 1 was administered to both Samples. Scoring was the same as in Study 1. Internal reliability for the new sample was good, ∝ = .81.

*Co-parenting communication*. We created binary items to capture respondents’ ability to communicate effectively with their ex-partner regarding childcare arrangements. These items focused on staying calm and communicating clearly. As such, the items we created were: ‘Mostly, I can manage my emotions when I'm in contact with my ex.’ and ‘Mostly, I feel able to calmly express my views about childcare arrangements to my ex.’ These items were answered yes/no.

*Psychological distress*. The 4-item Patient Health Questionnaire (PHQ4) [76] was used to measure psychological distress. The PHQ4 is a very brief measure of anxiety (2 items) and depression (2 items). Respondents rate the frequency with which they have experienced each of the four symptoms described in the preceding 2 weeks (e.g., ‘Feeling nervous, anxious, or on edge’, 0 = *not at all*, to 3 = *nearly every day*). Item responses are summed to give a total score. Reliability of the PHQ4 is good, ∝ = .85 [76].

*Stress*. The short form (4 item) Perceived Stress Scale (PSS) [77] was used to measure stress. The PSS measures the extent to which respondents feel their lives are uncontrollable, unpredictable, and overloading. The 4 item PSS has good psychometric properties [78]. Respondents rate the extent to which they have experienced the feelings described (e.g., ‘In the last month how often have you felt you were unable to control the important things in your life?’ 0 = *never*, to 4 = *very often*). Item responses are summed to give a total score. Reliability of the PSS-4 is good, ∝ = .77 [78].

### Study 2 results and discussion

Descriptive statistics can be found in Table 3.

#### Construct validity

*Convergent validity*. In Sample 3, (*n* = 156), the EARDA total score was significantly negatively correlated with both psychological distress, (*r* = -.56, *p* < .0001) and stress (*r* = -.55, *p*< .0001), such that individuals low in emotional adaptation tended to have high psychological distress and stress. Additionally, emotional adaptation was positively correlated with time since break up (*r =* .41, *p* < .001). In Sample 1, emotional adaptation correlated negatively with attachment anxiety (*r* = -.441, *p* < .0001), indicating that those with high attachment anxiety were likely to have low emotional adaptation. These findings provide support for the convergent validity of the new EARDA scale.

*Discriminant validity*. In Sample 1, emotional adaptation correlated negatively with attachment avoidance (*r* = -.153, *p* < .0001), indicating that those scoring high in attachment avoidance were likely to have low emotional adaptation, but importantly, the relationship is relatively weak, supporting the divergent validity of the EARDA.

#### Concurrent criterion-related validity

For the two co-parenting items, participants who responded positively had significantly higher emotional adaptation, supporting the criterion-related validity of the new scale (Table 4).

**Table 4:** *t*-tests for binary co-parenting items in Sample 3.

	EARDA score		
	No	Yes	*t*
Manage emotions	*N* = 41	*N* = 109	*t* (148) = -3.65**
	*M* = 50.61	*M* = 63.64	
	(SD 20.06)	(SD 19.26)	
Calmly express views about child care arrangements	*N* = 50	*N* = 100	*t* (148) = -2.67*
	*M* = 53.97	*M* = 63.14	
	(*SD* 21.75)	(*SD* 18.86)	

* *p* < .05

** *p* < .001.

#### Incremental validity

To examine incremental validity, using Samples 1 and 3, we examined whether emotional adaptation to relationship dissolution predicts effective co-parenting outcomes over and above previously identified predictors (i.e., attachment insecurity in Sample 1, and stress, and psychological distress in Sample 3). Specifically, we looked at co-parenting ability: managing emotions, and communicating calmly (Sample 3); and co-parenting conflict (Sample 1). In our data we have identified gender differences in who initiated the break up, where the child(ren) live, and levels of emotional adaptation to relationship dissolution Previous research has identified effects of age on attachment style [79], and stress and coping mechanisms [80]. In all adjusted analyses, therefore, demographic variables (age, gender) were entered in the first step. In the adjusted models, we also control for stress and distress for managing emotions and communicating calmly, as these are indicators of impairment to general psychological wellbeing/emotional state, and attachment insecurity for co-parenting conflict, as a reflection of relationship-related affect regulation strategies known to affect conflict resolution strategies [81]. We first report the unadjusted effect of emotional adaptation on effective co-parenting outcomes via simple logistic (managing emotions, and communicating calmly, in Sample 3) and linear (co-parenting conflict, in Sample 1) regression, and then report the adjusted effect via logistic and multiple hierarchical linear regression respectively.

To first examine the unadjusted effect of emotional adaptation to relationship dissolution on effective co-parenting outcomes in Sample 3, we conducted simple logistic regressions with only emotional adaptation to relationship dissolution as a predictor and the ‘managing emotions’ and ‘calmly express views’ variables as outcomes. For both analyses the model was a good fit, (managing emotions, χ^2^(1) = 12.45, *p* < .001; calmly express views, χ^2^(1) = 6.89, *p* = .009), and emotional adaptation was a significant predictor of participants ability to manage their emotions and calmly express their views when communicating with their ex-partner (Table 5).

**Table 5:** Unadjusted and adjusted logistic regressions predicting yes responses to managing emotions and calmly expressing views in Sample 3.

	Manage emotions					Calmly express views				
	B	S.E.	Wald	*P*	OR	B	S.E.	Wald	*p*	OR
Unadjusted										
EARDA score	.03	.01	11.35	.001	1.03	.02	.01	6.58	.01	1.02
Step 1 –adjusted										
Age	-.03	.02	2.15	.142	0.96	-.01	.02	0.06	.79	0.99
Gender										
Female	.40	.39	1.06	.30	1.50	.14	.36	0.16	.68	1.15
Step 2 –adjusted										
Age	-.04	.02	3.10	.07	0.95	-.01	.02	.12	.73	0.99
Gender										
Female	.54	.41	1.69	.19	1.71	.19	.36	.27	.60	1.21
Psychological distress	-.04	.07	0.45	.50	0.95	-.00	.06	.00	.96	0.99
Stress	-.19	.08	5.30	.02*	0.82	-.11	.07	2.19	.14	0.89
Step 3—adjusted										
Age	-.05	.02	4.16	.04	0.94	-.01	.02	0.34	.55	0.98
Gender										
Female	.79	.44	3.24	.07	2.21	.36	.38	0.35	.34	1.43
Psychological distress	.01	.07	0.01	.92	1.01	.04	.07	0.33	.56	1.04
Stress	-.15	.09	2.80	.09	0.86	-.06	.08	0.75	.38	0.93
EARDA score	.03	.013	5.06	.02	1.03	.02	.01	4.21	.04	1.02

To examine the adjusted effects, we carried out further logistic regressions with additional predictors. After entering demographics (age, *p* = .15, and gender, *p* = .47) at Step 1, we entered psychological distress and stress at Step 2. The model was a good fit, χ^2^(4) = 17.77, *p* = < .001, and indicated that stress was a significant predictor of participants’ ability to manage their emotions (*OR =* .*82*, *p* = .02); however, psychological distress was not (*OR =* .*89*, *p* = .14). Emotional adaptation to relationship dissolution was added at Step 3 and represented a significant improvement to the model, χ^2^(5) = 22.99, *p* = < .001, and whilst emotional adaptation was a significant positive predictor of managing emotions, stress no longer was (*p* = .10). This indicates that emotional adaptation predicts one’s ability to manage their emotions when communicating with an ex, over and above stress (Table 5).

A logistic regression was then carried out to examine the adjusted effect of emotional adaptation to relationship dissolution on the ability to calmly express views when communicating with an ex-partner. After entering age (*p* = .82) and gender (*p* = .91) at Step 1, we added psychological distress and stress at Step 2. The model was not a good fit, χ^2^(4) = 4.31, *p* = .365, and indicated that none of the variables included were significant predictors of the outcome variable. Emotional adaptation was added at Step 3, and represented a significant improvement to the model, χ^2^ (1) 4.10, *p* = .04, with emotional adaptation predicting one’s ability to calmly express their views when communicating with an ex, over and above the other variables included in the analysis (Table 5). However, the poor predictive power of the Step 2 variables was not overcome in Step 3, and the model remained a poor fit, χ^2^(5) = 8.42, *p* = .13. Of note, we repeated the two adjusted logistic regression analyses (for managing emotions and calmly expressing views) using a categorical recode of EARDA as low, medium, and high (based on cut offs from the bottom quartile, middle half, and upper quartile of Sample 1’s EARDA). We describe this in Study 4, where we apply the EARDA in a real world setting.

We then employed a simple linear regression with emotional adaptation to relationship dissolution predicting co-parenting conflict in Sample 1. The model was a good fit, *F*(1, 571) = 13.41, *p* < .001, and explained 2% of the variance in co-parenting conflict (Table 6). To examine the adjusted effect of emotional adaptation to relationship dissolution on co-parenting conflict, with attachment anxiety and avoidance as additional predictors, we employed a hierarchical regression to identify whether EARDA scores in Sample 1 could predict co-parenting conflict (QCCS conflict subscale) over and above attachment anxiety and avoidance (Table 6). Demographics were entered at Step 1. Gender explained a significant amount of variance in conflict, with conflict more likely in women (*M* = 11.18, *SD* = 0.22) compared to men (*M* = 10.06, *SD* = 0.30). Attachment anxiety and avoidance were entered at Step 2. The model was a good fit (*p* = .02) and gender was a significant predictor of conflict (*ß* = .1.04, *p* = .01). Attachment anxiety (*ß* = .34, *p* = .005), but not avoidance, also significantly predicted variance in co-parenting conflict, with higher anxiety associated with increased co-parenting conflict. Emotional adaptation to relationship dissolution was entered at Step 3, and there was significant model improvement compared to Step 2 (sig. *F* change *p* = .009). Higher emotional adaptation was significantly associated with lower conflict (*ß* = -0.02, *p* = .009).

**Table 6:** Multiple regressions predicting co-parenting conflict in Sample 1.

Predictor	*t*	*p*	*ß*	*F*	*df*	*p*	*adj R*^*2*^	*R*^*2*^ *chg*
*Unadjusted model*				13.41	1, 571	< .001	.023	
EARDA score	-3.66	< .001	-.15					
*Adjusted model*								
Step 1				4.15	2, 570	.016	.011	.014*
Age	0.58	.55	.013					
Gender	2.87	.004	1.18					
Step 2				4.44	4, 568	.002	.024	.015*
Age	1.04	.29	.023					
Gender	2.54	.01	1.04					
Attachment Anxiety	2.80	.005	0.34					
Attachment Avoidance	0.93	.35	0.12					
Step 3				4.97	5, 567	< .001	.035	.012*
Age	0.89	.37	0.01					
Gender	2.77	.006	1.13					
Attachment Anxiety	1.35	.17	0.18					
Attachment Avoidance	0.62	.53	0.08					
EARDA score	-2.62	.009	-0.02					

*p < .05.

The findings from these three analyses suggest that emotional adaptation to relationship dissolution is a predictor of the ability to co-parent effectively, and co-parenting conflict, over and above psychological distress and stress, and attachment anxiety, respectively. In addition, the tests of convergent, discriminant, and concurrent criterion-related validity provide further support for the construct validity of the EARDA for measuring emotional adaptation to relationship dissolution. The EARDA demonstrated the expected moderate correlations with stress, distress, and attachment anxiety (*r* ≥ .30), and a small association with attachment avoidance (*r* < .20). Scores on the EARDA also distinguished those who responded positively versus negatively to the co-parenting items, in line with our model of emotional adaptation to relationship dissolution, which posits co-parenting quality as an expected outcome. Having found support for the convergent, discriminant, criterion-related validity, and incremental predictive validity of the EARDA, we next assess two further key aspects of construct validity, namely the nomological validity emotional adaptation to relationship dissolution, and the generalizability of the construct validity using a different population.

## Study 3

The EARDA was developed to address the issue of how low emotional adaptation to relationship dissolution might impede individuals’ capacity to effectively engage with the pragmatic complexities of their separation, including co-parenting. The literature suggests that following separation, continued contact with an ex-partner (as in the case when there are children) can result in any negative affect felt about the relationship and break-up being maintained [23]. Because relationship dissolution, and therefore emotional adaptation and readiness to deal with the pragmatics of a separation are likely to be experienced differently among those who have and don’t have children, we examined the EARDA in a mixed parent and non-parent sample. Accordingly, we examined the relevance of the construct of emotional adaptation to relationship dissolution for a mixed sample of parents and non-parents, because the ability to ‘resolve’ one’s emotional reaction to a break-up (i.e., achieve emotional adaptation) may be different in couples who have children and those who do not. Testing the generalizability of the EARDA across different populations this way also provides an elaboration of its construct validity [61]

To further establish the construct validity of the new emotional adaptation to relationship dissolution construct, we also sought to locate emotional adaptation to relationship dissolution within the network of other potentially related and unrelated variables. Constructing this *nomological network* for the construct allowed us to embed it within the network of other variables that theoretically should be positively, negatively, or not at all related to it [46]. Establishing the nomological network of emotional adaptation to relationship dissolution can be viewed as an extension of the preliminary tests of convergent and discriminant validity, by situating it within a broader network of constructs to better understand its meaning.

Tests of this nomological validity are most commonly accomplished by examining the correlations of the new construct with a range of other constructs. We supplemented these basic correlational tests of the nomological network with a hierarchical cluster analysis and multidimensional scaling to obtain a more complete profile, and thus understanding, of emotional adaptation to relationship dissolution and its meaningful associations with relevant constructs. Several different personality constructs, coping styles, emotion regulation and emotional intelligence were chosen to test the nomological network of emotional adaptation to relationship dissolution using these techniques. Drawing from the assumptions of our model, we expected emotional adaptation to relationship dissolution to be positively associated with adaptive coping (e.g., acceptance), outward focussed personality traits (e.g., extraversion), and psychological flexibility, whilst being negatively associated with maladaptive coping strategies (e.g., denial) and inward focussed responses (e.g., perfectionism).

### Study 3 method

#### Participants and procedure

Data from Sample 2 were analysed for Study 3 (see Study 1 for details).

#### Measures

Cronbach’s alphas for all the measures presented in this section in Samples 2 can be found in Table 7.

**Table 7:** Descriptive statistics and gender differences for all variables included in cluster and MDS analyses for Sample 2.

Variable	Overall M (SD)	α	Men M (SD)	Women M (SD)	Gender diff. *t* test
Emotional Adaptation to Relationship Dissolution	64.93 (21.02)	.82	61.30 (20.53)	66.34 (21.14)	*t*(192) = -1.46, *p* = .14
Acceptance	3.58 (1.20)	.88	3.57 (1.27)	3.59 (1.18)	*t*(173) = -0.14, *p* = .89
Emotional intelligence	3.32 (0.93)	.85`	3.57 (1.02)	3.27 (0.86)	*t*(183) = 2.04, *p* = .04*
Attachment anxiety	4.37 (1.60)	.89	4.03 (1.74)	4.46 (1.52)	*t*(180) = -1.60, *p* = .11
Socially prescribed perfectionism	3.64 (1.31)	.77	3.80 (1.20)	3.54 (1.30)	*t*(184) = 1.25, *p* = .21
Active coping	2.83 (0.79)	.71	2.78 (0.70)	2.86 (0.82)	*t*(194) = -0.59, *p* = .56
Acceptance coping	2.68 (0.75)	.42	2.76 (0.74)	2.66 (0.75)	*t*(194) = 0.78, *p* = .44
Behavioural disengagement	1.57 (0.71)	.61	1.64 (0.68)	1.53 (0.69)	*t*(191) = 0.98, *p* = .33
Denial	1.47 (0.73)	.71	1.58 (0.72)	1.41 (0.71)	*t*(191) = 1.43, *p* = .16
Self-blame	2.50 (0.89)	.74	2.51 (0.79)	2.49 (0.92)	*t*(194) = 0.16, *p* = .87
Substance use	1.67 (0.90)	.95	1.73 (0.95)	1.63 (0.86)	*t*(193) = 0.66, *p* = .51
Agreeableness	3.03 (0.64)	.74	3.19 (0.56)	2.97 (0.65)	*t*(190) = 2.12, *p* = .04*
Conscientiousness	3.63 (0.62)	.75	3.46 (0.74)	3.70 (0.57)	*t*(189) = -2.32, *p* = .02*
Extraversion	3.15 (0.69)	.78	3.14 (0.59)	3.18 (0.70)	*t*(190) = -0.40, *p* = .69
Honesty/ humility	3.47 (0.64)	.69	3.36 (0.65)	3.50 (0.63)	*t*(188) = -1.32, *p* = .19

*Demographics*. Participants completed measures that recorded information about their relationship with their ex-partner as outlined in Study 1 of this paper.

*Emotional adaptation to relationship dissolution*. The Emotional Adaptation to Relationship Dissolution Assessment (EARDA) developed for the current set of studies and outlined in Study 1 was used.

*Coping*. The BriefCOPE [82] was used to measure participants’ dominant coping mechanisms when experiencing stress. The BriefCOPE is a 28-item self-report measure that assesses 14 dimensions of coping using two item subscales (αs reported from Carver [82]): self-distraction (α = .71), active coping (α = .68), denial (α = .54), substance abuse (α = .90), emotional support (α = .71), instrumental support (α = .64), behavioural disengagement (α = .65), venting (α = .50), positive reframing (α = .64), planning (α = .73), humour (α = .73), acceptance (α = .57), religion (α = .82), and self-blame (α = .69). Participants were instructed to think about how they deal with stress generally in their life and to rate how much they used that particular coping mechanism on a 4 point Likert scale (1 = *I don’t do this at all*, to 4 = *I do this a lot*). Mean scores for each subscale were calculated with higher scores indicated greater use of that particular coping mechanism.

*Forgivingness*. Participant’s trait forgiveness was measured using the Tendency to Forgive Scale (TFF) [83]. The TTF is a 10-item scale that assessed participants’ overall forgivingness and consists of two subscales: tendency to forgive (e.g., “I tend to get over it quickly when someone hurts my feelings”) and attitudes towards forgiveness (“It is admirable to be a forgiving person”). Participants were asked to rate items on a 6-point Likert scale (1 = *Strongly disagree*, 6 = *Strongly agree*). Mean scores were calculated as a single scale, with negatively worded items reverse coded, and higher scores indicated greater trait forgiveness. Reliability for the TTF is good, α = .81 [83].

*Personality*. The HEXACO-60 [84] was used to measure participants’ personality structure. The HEXACO-60 consists of six 10-item subscales that measure the 6 dimensions of the HEXACO model of personality (α reported on community sample) [84], humility-honesty (“I wouldn’t use flattery to get a raise or promotion at work, even if I thought it would succeed”; α = .74), emotionality (“I would feel afraid if I had to travel in bad weather conditions”; α = .73), extraversion (“I feel reasonably satisfied with myself overall”; α = .73), agreeableness (“I rarely hold a grudge, even against people who have badly wronged me”; α = .75), conscientiousness (“I plan ahead and organize things, to avoid scrambling at the last minute”; α = .76), and openness to experience (“I’m interested in learning about the history and politics of other countries”; α = .80). Participants were asked to rate items on a 5 point Likert scale (1 = *Strongly disagree*, 5 = *Strongly agree*) and mean scores were calculated for each subscale with negatively worded items reverse coded.

*Emotion Regulation (ERQ)*. The Emotion Regulation Questionnaire (ERQ) [85] was used to assess emotion regulation strategies using two 6 –item subscales: cognitive reappraisal (e.g., “I control my emotions by changing the way I think about the situation I’m in” α = .79) and expressive suppression (e.g., “I control my emotions by not expressing them” α = .73). Participants were asked to rate items on a 7 point Likert scale (1 = *Strongly disagree*, 7 = *Strongly agree*) and mean scores for each subscale were calculated.

*Trait acceptance/ psychological flexibility*. The 7-item Acceptance and Action Questionnaire (AAQ-II) [86] was used to assess participants’ trait acceptance. The AAQ-II measures acceptance as psychological flexibility in stressful situations and participants were asked how much the agreed with each statement on a scale of 1–7 (1 = *Strongly disagree*, 7 = *Strongly agree*). Mean scores were calculated as a single scale and higher scores indicated greater psychological flexibility. Reliability for the AAQ-II is good, α = .88 [86].

*Perfectionism MPS-SF*. Trait perfectionism was measured using the Multidimensional Perfectionism Scale–Short Form (MPS-SF) [87]. The MPS-SF measures perfectionism across three five item subscales: self-oriented perfectionism (“One of my goals is to be perfect in everything I do”, α = .86); other-oriented perfectionism (“It does not matter when someone close to me does not do their absolute best”, α = .82), and socially prescribed perfectionism (“Anything I do that is less than excellent will be seen as poor work by those around me”, α = .87). Participants were asked how much the agreed with each statement on a scale of 1–7 (1 = *Strongly disagree*, 7 = *Strongly agree*). Mean scores for each subscale were calculated with higher scores in the self-oriented and socially-prescribed subscales and lower scores in the other-oriented subscale indicating greater perfectionism. Reliability for the MPS-SF range from α = .75-.85 [88].

*Emotional intelligence*. The Survey of Emotional Intelligence (SEI) [89] was used to measure participant’s regulation of emotion in the self. Sample items include “Sometimes my moods get the best of me” (reverse coded) and “I am capable of staying under control during an argument”. Participants were asked to rate their agreement with each statement on a 6-point Likert scale (1 = *Disagree very much*, 6 = *Agree very much*). Mean scores were calculated (negatively worded items were reverse coded) with higher scores indicating greater emotional intelligence. Reliability is good for the SEI, α = .81 [89].

*Adult attachment*. Adult attachment was assessed using the ECR-12 [74] as described in Study 2 of this paper.

#### Analytic strategy

As the key aim of the current study is to examine the nomological network of the emotional adaptation to relationship dissolution construct, and initial correlation analysis was conducted to examine the association between emotional adaptation to relationship dissolution and all of the variables included in the questionnaire. As the aim of the current study is to examine how emotional adaptation is related to other potential constructs within its nomological network, only non-demographic variables that had a significant association with emotional adaptation were included in the cluster analysis and multidimensional scaling (MDS).

A hierarchical cluster analysis with Ward’s procedure (squared Euclidian distances between participants) was conducted on emotional adaptation to relationship dissolution and the constructs identified as significantly related to it. As Euclidian distance techniques are sensitive to variations in scale measurement (and outliers) all data were transformed into z-scores [90]. Multidimensional scaling using the PROXSCAL method was employed on the z-transformed variables included in the cluster analysis. Although originally developed to measure dissimilarity assessments by independent raters, it has been identified as a useful tool for discovering the dimensionality that underlies participants’ response data and for evaluating consistency of responses across groups [91].

A dual approach to determining the nomological network of emotional adaptation to relationship dissolution (cluster analysis alongside MDS) was chosen to assess construct equivalence through examining similarity and dissimilarity to relevant related constructs. By demonstrating the nomological network of emotional adaptation to relationship dissolution we are able to identify those variables with which it shares a similar dimensional theoretical space and those with which it is distant.

### Study 3 results and discussion

#### Descriptive statistics

All variables were tested and found to be in the acceptable range of normality for the planned analyses. Descriptive statistics and gender differences are presented in Table 7, and demonstrate a significant difference between men and women in emotional intelligence, agreeableness, and conscientiousness.

#### Correlations

**Fig 2:**
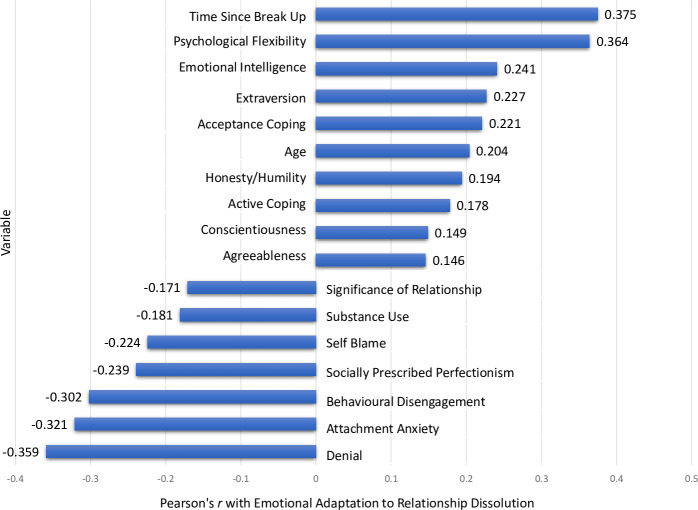
Correlations of emotional adaptation to relationship dissolution with validity variables.

Correlation analysis suggests that emotional adaptation to relationship dissolution is significantly correlated with the variables presented in Fig 2. This initial analysis demonstrates the positive association between emotional adaptation and positive coping strategies, outward focused traits, and psychological flexibility, as well as time since break up. There is also a clear negative association between emotional adaptation to relationship dissolution and negative coping strategies and inward focused traits, as well as significance of the relationship. Emotional adaptation to relationship dissolution was not significantly associated with the coping dimensions of self-distraction, emotional support, instrumental support, venting, positive reframing, planning, humour, or religion. Emotional adaptation to relationship dissolution was also not associated with forgiveness, emotionality, openness to experience, cognitive reappraisal, expressive suppression, attachment avoidance, self-oriented perfectionism, or other-oriented perfectionism.

#### Cluster analysis and multidimensional scaling

**Fig 3:**
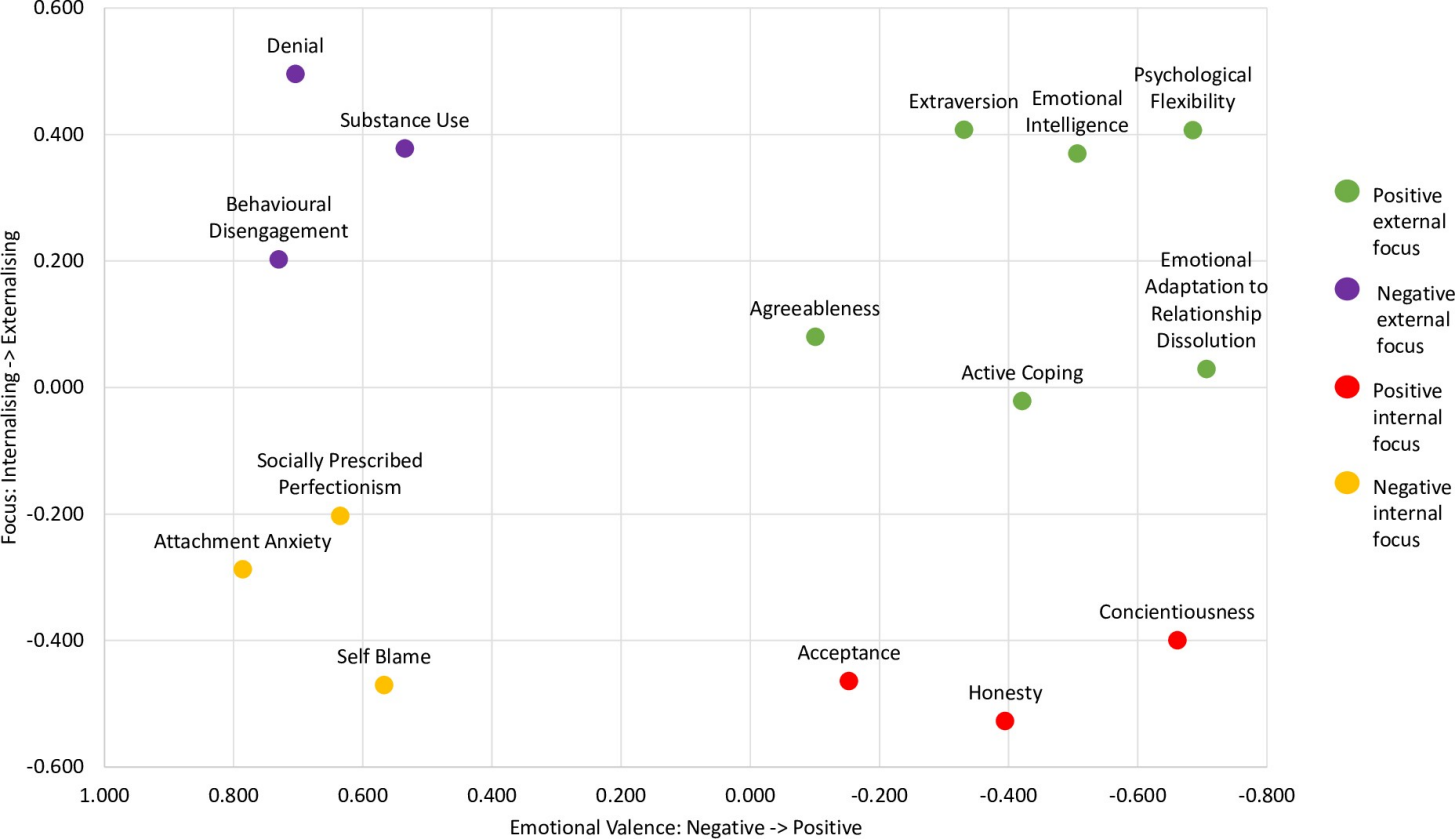
Emotional adaptation to relationship dissolution multidimensional scale.

The results from the cluster analysis suggest the variables most closely associated with emotional adaptation to relationship dissolution are best conceptualised as falling into four clusters (Table 8). *Psychological and emotional flexibility* is typified by responses and traits that indicate an outward focussed and active responsiveness; *positive adaptation* consists of variables that suggest positive adaptive strategies, both state and trait; *maladaptive coping strategies* consists of variables which although used as coping strategies, may result in negative effects for self and other; finally, *negative self model* consists of variables which suggest a negative internalised model of self. Amongst these clusters, emotional adaptation to relationship dissolution fits into the positive adaptation category. The results of the MDS (Fig 3) suggest two dimensions which may underlie these clusters: valence of emotion (positive, negative) and internal working model (model of world, model of self). The results of the MDS suggest that emotional adaptation to relationship dissolution is most distinct from attachment anxiety (Euclidian distance = 1.53) and most similar to active coping (Euclidian distance = .290).

**Table 8:** Clusters identified in cluster analysis.

Cluster name	Relevant constructs
Psychological and emotional flexibility	Psychological flexibility
	Emotional intelligence
	Active coping
	Extraversion
	Conscientiousness
Positive Adaptation	Honesty
	Agreeableness
	Acceptance based coping
	Emotional adaptation to relationship dissolution
Maladaptive coping strategies	Substance use
	Behavioural disengagement
	Denial
Negative self-model	Anxiety
	Self-blame
	Socially prescribed perfectionism

Sensitivity analyses were carried out to check whether there were differences in emotional adaptation to relationship dissolution between parents and non-parents in this sample, and whether the nomological network of emotional adaptation varied as a function of being a parent. An independent t-test was conducted that demonstrates no significant difference in emotional adaptation between parents and non-parents in this sample, *t*(195) = .20, *p* = .84. Cluster analysis (Ward’s method) on the non-parent sample only indicate no difference in clusters identified when compared to the mixed sample of parents and non-parents. Finally, Multidimensional Scaling (PROXSCAL) produce the same dimensions (focus and emotional valence) as the mixed parent/ non-parent sample. These findings indicate that in the current study the nomological network of emotional adaptation to relationship dissolution did not differ as a function of parenthood status.

Together, the results from the cluster analysis and MDS provide good preliminary evidence of the nomological network of the emotional adaptation to relationship dissolution construct. In line with the assumptions underlying our theoretical model, emotional adaptation to relationship dissolution was located in close proximity to active coping, and adaptive coping strategy, and furthest away from self-blame, a maladaptive coping strategy. Emotional adaptation was also positively associated with individual differences associated with positive emotions, having an outward focus, and psychological flexibility (e.g., extraversion, agreeableness) and negatively associated with individual differences reflecting negative emotions and an inward focus (e.g., socially prescribed perfectionism and anxiety). Furthermore, the correlations show that emotional adaptation to relationship dissolution was not associated with constructs with which it is conceptually unrelated.

## Study 4

Having established that the EARDA is a reliable and unidimensional scale (Study 1), with good construct validity (Study 2), and demonstrates meaningful relationships with related constructs (Study 3), in Study 4 we take a triangulated approach and compare EARDA scores against expert professional opinion. The purpose of this is twofold. Firstly, we aim to further validate the EARDA by moving beyond self-report, and seeing whether EARDA scores tally with the opinions of third party observers (in this case, mediators). Here, we supplement our previous assessment of concurrent criterion validity (where we found that participants with higher EARDA scores had better co-parenting communication, study 2) by examining the relationship between EARDA scores and mediators’ assessments of readiness to make childcare arrangements. Secondly, we seek to assess the feasibility of establishing cut-off scores that could be used in applied settings, for example, to triage to different levels of support in the various pathways to family justice [51].

### Study 4 method

#### Participants and procedures

Two mediators administered the EARDA to *N* = 30 clients who were separated parents undergoing an initial mediation information session (Mediation Information Assessment Meeting). MIAMs are compulsory for those applying for court proceedings to settle separation disputes in England and Wales. Separated parents seeking MIAMs can therefore be considered en-route to court proceedings, unless the MIAM results in a decision to use mediation instead of court proceedings to settle their dispute. Ethical approval for the study was received from The University of Sheffield Ethics Committee (Psychology) prior to data collection, and all participants (clients and mediators) provided informed consent. Basic demographic information was collected about clients, such as their age, gender, and how many children they had. Clients were asked by their mediators to complete the EARDA, mediators and clients then completed the single-item measures described below. Mediators were blind to EARDA responses at the point that they completed their measures.

#### Measures

*Demographics*. Data on age, gender, number of children, and time since break up was collected for clients in the validity study.

*Emotional adaptation to relationship dissolution*. The EARDA developed for the current set of studies and outlined in Study 1 of this paper was used. In order to propose preliminary cut-off categories, EARDA scores were recoded as low, medium, or high based on the lower quartile (0-< 40), middle half (40-< 74), and upper quartile (74–100) of EARDA scores of participants in Sample 1. This sample was selected as it is the largest sample (*n* = 573), in which time since separation was < 6 years, likely providing more useful cut-offs than a broader range (such as that in Sample 2). These cut-offs were applied in supplementary analyses that replicate the adjusted logistic regression models presented in Study 2 (Table 5). EARDA was entered as a categorical predictor, using the cut offs specified above. Findings indicated that separated parents with high and medium emotional adaptation to relationship dissolution had 4.16 and 1.80 times the odds of reporting that they could (vs. could not) manage their emotions when communicating with their ex-partner than those with low emotional adaptation, respectively. Those with high and medium emotional adaptation also had 3.15 and 2.44 times the odds of reporting that they could (vs. could not) calmly express their views when communicating with their ex-partner than those with low emotional adaptation, respectively. Taken together, the findings lend support to the utility and meaningfulness of these cut offs for identifying different levels of emotional adaptation.

*Single item validity measures*. Mediators were asked to indicate how ready they perceived their client to be to make child-care arrangements with their ex-partner without having arguments, using a categorical response of low, medium, or high. Clients were asked to self-report to an equivalent question using the same low, medium, and high categories. Mediators were also asked how confident they were in their decision (1 = *not at all confident*, 5 = *very confident*).

### Study 4 results and discussion

#### Descriptive statistics

Six participants were excluded from analysis due to mediator confidence being ≤ 3 (‘Not sure’). One outlier was excluded, as their emotional adaptation to relationship dissolution score was in the top quartile (82%) whilst their mediator assessment was in the low-readiness category and they were the only participant to show this pattern. The remaining participants were 12 females, 10 males, and 1 transgender female, aged 22 to 50 (*M* = 36.35, *SD* = 7.86). There were no significant differences in age between male and female participants, t(20) = .26, *p* = .80. reliability for the EARDA was good in the current sample, α = .83.

The number of children participants had ranged from 1 to 3 (*M age* = 7.92, *SD age* = 4.22). When asked how ready participants felt to make child-care arrangements with their child(ren)’s other parent without having arguments, 9 (39.1%) reported feeling able, 11 (47.8%) reported needing some support, and 3 (13%) reported needing help to manage their emotions. Time since the relationship break up was communicated to both partners ranged from 2 months to 10.5 years (*M* = 2.43, *SD* = 2.38). When asked who initiated the separation, 7 (30.4%) of participants reported that they had, 8 (34.8%) that their ex-partner had, 6 (26.1%) reported that it was mutual, and 1 (4.3%) reported that they would rather not say. A chi square indicated that these frequencies did not differ by gender (χ^2^(6) = 4.61, *p* = .60).

#### Correlations

Pearson’s product-moment correlation analysis indicated that there was a significant, strong association between the low, medium and high emotional adaptation to relationship dissolution categories and mediators assessment of readiness to engage in co-parenting with their ex-partner, *r* = .71, *p* < .001. There was a significant, moderate correlation between participants own assessment of emotional readiness and the previously described emotional adaptation to relationship dissolution categories, *r* = .49, *p* = 02. That the EARDA cut offs proposed here are strongly associated with professional assessment of separating parents’ readiness to make child-care arrangements with their child's other parent without arguing, and moderately associated with separating parents’ own response to the same question, suggests that the EARDA categories are a closer match to a highly trained, objective, third party observer’s opinion than single item self-report. This might suggest that the combined data capture of the EARDA 10 items is accessing something more closely aligned with objective measurement, and less closely aligned with consciously controlled self-report. However, the sample size is small, and the assessment is limited to a single, specific domain: the readiness to make child-care arrangements without arguing. Further research is needed to examine whether these cut offs would also correlate with professional opinion in related areas, such as co-parenting support. That said, the cut offs proposed show promise, and could offer practitioners a way to usefully triage clients. Having established that when using low, medium, and high cut-offs, the EARDA correlates highly with expert opinion of co-parenting readiness, next, we revisited our original model of emotional adaptation to relationship dissolution and set out to test some proposed pathways within it using mediation analysis.

## Study 5

In our model of emotional adaptation to relationship dissolution, we proposed it to be a key predictor of post-relationship dissolution outcomes, including co-parenting, and affected by factors concerning the nature of the separation (Assumption 1). In other words, we can view emotional adaptation to relationship dissolution as a mediator of the relationship between contextual, separation variables, and co-parenting. In Study 5, we test these aspects of our model using mediation analysis. We examine whether emotional adaptation to relationship dissolution mediated the associations between predictors of co-parenting quality (namely time since separation, who initiated the break up, and whether it was a shock or not), and co-parenting quality indicators (co-parenting conflict and support). Analysis was conducted using data from Sample 1, described in Study 1 of this paper.

### Method

#### Procedure

PROCESS (model 4) mediation analysis [92] was employed to test whether emotional adaptation to relationship dissolution mediated the relationship between factors concerning the nature of the separation (time since break up, whether the break up was a shock, and who initiated the break up) and the co-parenting dimensions of conflict and support. As such, 6 mediation models were conducted with emotional adaptation to relationship dissolution as the mediator.

#### Measures

The following measures were used in this analysis, all as described in Study 1: Quality of Co-parental Communication, Conflict and Support sub-scales [72], EARDA, time since break up, shock associated with break up, and who initiated the break up.

### Study 5 results and discussion

There was no direct or indirect effect of time, shock, or who initiated break up on co-parenting support. However, for co-parenting conflict, there was a significant indirect effect, via emotional adaptation to relationship dissolution, of time, shock, and who initiated the breakup, on co-parenting conflict. The results, presented in Table 9, suggest that emotional adaptation fully mediates the association between time and co-parenting conflict—the greater the amount of time since break up, the higher the emotional adaptation, and this accounts for lower levels of co-parenting conflict. There is a direct effect of who initiated the breakup on co-parenting conflict, with less conflict present for those who made a mutual decision to separate, and an indirect effect, via emotional adaptation, for those whose ex-partner initiated the separation. Here, low emotional adaptation to relationship dissolution accounts for high conflict, among those whose ex-partner initiated the separation. Finally, emotional adaptation fully mediated the association between shock about the breakup and conflict. Those who were either not shocked, or were not sure if they were shocked, experienced higher emotional adaptation and in turn lower co-parenting conflict, than those who were shocked.

**Table 9:** Effects of emotional adaptation to relationship dissolution on the relationship between relationship dissolution features and co-parenting conflict.

		Co-parenting conflict	*R*^*2*^
Independent variable	Direct and indirect effects	*B* (95% CI)	
Time			
	Direct effect	.09 (-.15, .33)	
	Indirect effect via Emotional adaptation to relationship dissolution	-.10* (-.17, -.04)	.02*
Initiation of break up			
	Direct effect		
	ex- initiated vs self-initiated	-.37 (-1.26, .51)	
	`mutual vs. self-initiated	-1.97 (-2.86, -1.08)*	.05*
	rather not say vs. self-initiated	-1.67 (-4.9, 1.57)	
	Indirect effect via Emotional adaptation to relationship dissolution		
	ex- initiated vs self-initiated	.45 (.18, .75)*	
	mutual vs. self-initiated	.09 (-.04, .28)	
	rather not say vs. self-initiated	.25 (-.31, .89)	
Shock about break up			
	Direct effect		
	not shocked vs. shocked	-.39 (-1.29, .50)	.02*
	not sure vs. shocked	-1.23 (-3.21, .75)	
	Indirect effect via Emotional adaptation to relationship dissolution		
	not shocked vs. shocked	-.53 (-.91, -.18)	
	not sure vs. shocked	-.46 (-.89, -.12)	

*p <. 05.

The findings from this study are consistent with the factors theorised to contribute to the development of emotional adaptation to relationship dissolution as well as its proposed outcomes.

## General discussion

In this paper, we have presented the new construct of emotional adaptation to relationship dissolution after relationship dissolution, proposed a model that details its processes and locates it within relevant theories of coping and grief, and developed and tested the Emotional Adaptation to Relationship Dissolution Assessment, for use by future researchers and practitioners. Here, we discuss how our data support the model, the performance of the EARDA in multiple testing phases, and the theoretical and practical context of emotional adaptation to relationship dissolution. We also detail the implications of this work for policy and practice, and lay out a future research agenda for emotional adaptation to relationship dissolution.

### The construct of emotional adaptation to relationship dissolution

Emotional adaptation to relationship dissolution is a new construct that describes the process of adaptation to the dissolution of a relationship. It draws from the VSA model of couple adaptation to stress, and the DMP model of coping with grief, and is informed by Duck’s [17] and Lee’s [20] models of relationship dissolution, as well as theories of co-parenting. As such, the construct of emotional adaptation to relationship dissolution builds on existing theories but also moves beyond them, to explain the psychological processes that occur in between relationship dissolution and a range of relevant outcomes, including capacity for negotiation and co-parenting quality. Our construct of emotional adaptation to relationship dissolution is underpinned by several key assumptions that build on the above theories, and that we tested across the five studies. We have proposed that emotional adaptation to relationship dissolution is a malleable but enduring, state-level construct, with a starting point at the point of relationship dissolution (Assumption 1), and that emotional adaptation increases over time (Assumption 2). Our data support this proposition, as emotional adaptation to relationship dissolution was positively associated with time since break-up in 4 different samples (Studies 1 to 3). The converse to this is that those who scored low on emotional adaptation to relationship dissolution were individuals who had most recently experienced a relationship breakup, highlighting that relationship dissolution is the starting point for the development of greater emotional adaptation.

Assumption 3 states that increases in emotional adaptation to relationship dissolution are associated with more positive emotions and fewer negative ones, and Assumption 4 proposes that the use of adaptive coping strategies facilitates increases in emotional adaptation to relationship dissolution over time. Although not longitudinal, our data offer initial support these assumptions. We found that stress and distress are lower in those with higher emotional adaptation to relationship dissolution (Study 2). Furthermore as a construct, emotional adaptation to relationship dissolution is linked to a cluster of variables that represent positive adaptation, and that are associated with positive emotions (e.g., extraversion, agreeableness, active coping, psychological flexibility), in a dimensional space representing views of self and views of the world (Study 3). The greater distance of emotional adaptation from variables reflecting maladaptive coping, and negative emotions (e.g., self-blame coping, and anxiety) provides additional support for these assumptions.

Our data also offer preliminary support for Assumption 5, that individual differences in enduring strengths and vulnerabilities will facilitate or hinder the transition from low to high emotional adaptation to relationship dissolution. In Study 2 (Sample 1), we found that both dimensions of attachment insecurity—avoidance and anxiety—were negatively related to emotional adaptation to relationship dissolution. Given that attachment insecurity is a trait-like variable that reflects negative views of the self, we conceptualise it as an individual difference vulnerability factor in our model. Attachment anxiety leads to ruminative preoccupation with relationships and the availability of loved ones [93] and as such this is highly likely to impede an individual’s movement from low to high emotional adaptation. Attachment avoidance, on the other hand, is characterised by denial and suppression of relationship-related emotions [93], rendering transition from low to high emotional adaptation via, active coping strategies, and fully processing emotional reactions, less likely. In addition, emotional adaptation to relationship dissolution was also associated with socially prescribed perfectionism in Study 2, an individual difference that has been shown to be a core vulnerability factor for poor mental health and other unhealthy outcomes [94]. Socially prescribed perfectionism, with its focus on trying to meet unrealistic standards set by others and society, and excessive rumination over failure [87], is also likely to impede progress towards emotional adaptation following a relationship break-up. Lastly, emotional adaptation to relationship dissolution was positively associated to individual differences well known to promote resilience in the face of stressors, and which have more of an outward versus inward focus, including emotional intelligence [95], honesty/humility [96], extraversion, and agreeableness [97].

While it was beyond the scope of the current paper to test Assumption 6 (that the transition from low to high emotional adaptation to relationship dissolution may follow a dynamic, non-linear pathway involving cycling back to lower levels of emotional adaptation) our future research agenda (below) seeks to address this.

### The nomological network of emotional adaptation to relationship dissolution

The results from Studies 3 to 5 situate emotional adaptation to relationship dissolution within a constellation of related and unrelated constructs to provide initial evidence of its nomological network. Using both basic (correlations) and more advanced (cluster analysis, MDS, mediation analysis) statistical techniques, we found that emotional adaptation to relationship dissolution demonstrated meaningful associations with a number of variables that it should be theoretically linked to, whilst showing appropriately low or no associations with variables with which it would be expected to have no linkage. For example, emotional adaptation to relationship dissolution was not significantly associated with forgiveness or attachment avoidance (among a mixed sample of parents and non-parents); however, it was significantly associated with emotional intelligence and attachment anxiety.

Although the distinctions between these pairs of apparently related variables–forgiveness and emotional intelligence, and anxious and avoidant attachment–may appear arbitrary, from a theoretical perspective the distinctions are both meaningful and important with respect to understanding the nature of emotional adaptation to relationship dissolution. Forgiveness involves the reduction of negative emotions, motivations and resentment towards a transgressor [98], whereas emotional intelligence involves the capacity to identify, express, understand and deal with emotions [99]. The latter captures the adaptive coping aspects of emotional adaptation to relationship dissolution and suggests that those high on emotional adaptation have a greater awareness of their emotional states and how best to manage them following relationship dissolution. With respect to forgiveness, we have defined the transition from low to high emotional adaptation as including not only the reduction of negative emotions, but also a cognitive shift from inward to outward focus, and a greater balance of positive emotions. Trait forgiveness, as measured by the Tendency to Forgive Scale (TTF) [83]) does not address these aspects of emotional adaptation the proposed emotional complexity involved. The lack of a significant correlation therefore confirms this assertion. Future research could measure state forgiveness, which typically assesses both a reduction in negative and an increase in positive cognitions, emotions, and behaviours [100] whereby we might expect to see a small positive correlation with emotional adaptation to relationship dissolution.

The differential associations with attachment anxiety and avoidant attachment provide further insights into the construct of emotional adaptation to relationship dissolution. Attachment avoidance and anxiety are dimensions of attachment insecurity, resulting from a history of received care that is rejecting or inconsistent, respectively [75]. However, while both dimensions of insecurity stem from suboptimal caregiving experiences, there are important differences between the two. Attachment anxiety reflects a hyperactivating affect regulation strategy, whereby negative emotions are amplified and sustained, ruminative focus on the availability of close relationship partners, and fears about abandonment [65]. The moderate negative association with emotional adaptation to relationship dissolution is consistent with our proposition that low emotional adaptation is characterised by predominantly negative emotions, and a loss and past focused orientation. Attachment avoidance, on the other hand, represents a deactivating affect regulation strategy, whereby negative emotional experiences, particularly those related to relationships, are supressed and denied, in favour of a compulsive self-reliance [65]. It is notable that in a mixed sample of parents and non-parents (Study 3), we found no association between avoidance and emotional adaptation to relationship dissolution, whereas in a sample of solely parents (Sample 1), we found a small negative association. This is likely to do with the continued contact and involvement that children bring to the separation process [23]. Given the opportunity, those high in avoidance are likely to simply cut off their relationship-related negative emotions. Parents who separate, however, are not given such an opportunity. For separating parents high in avoidance, continued contact with the ex-partner undermines their preferred coping strategies, thus potentially reducing their emotional adaptation to relationship dissolution.

In addition to establishing linkages between emotional adaptation to relationship dissolution and constructs proposed to be part of its nomological network, in Study 5, we also addressed the issue of the antecedents and consequences of emotional adaptation to relationship dissolution. Examining the antecedents and consequences of a construct are two important aspects of nomological validity which are often overlooked during scale development and validity testing [101]; hence, this is a notable strength of our work. That the links between time, shock, and who initiated the break up on co-parenting conflict were fully mediated by emotional adaptation to relationship dissolution provides good preliminary support for the hypothesised antecedents and consequences of the construct, minimising the chances that the bivariate relationships between the emotional adaptation and its proposed correlates reflect “illusory predictions” [101].

### The emotional adaptation to relationship dissolution assessment

Emotional adaptation to relationship dissolution bridges the gap between models of relationship dissolution and models of co-parenting. We have not only conceptualized this new construct but also developed a way of assessing it, offering a validated a scale that be used by other researchers and practitioners. The Emotional Adjustment to Relationship Dissolution Assessment (EARDA) is a 10-item. In Study 1, using two independent factor analyses, we showed that the EARDA is a unidimensional scale with good internal consistency. In Study 2, we demonstrated the convergent, discriminant, concurrent criterion-related, and incremental validity of the EARDA. The scale performed well. Our most noteworthy findings here were that: i) emotional adaptation predicts co-parenting ability over and above demographics, stress, and distress; and ii) emotional adaptation predicts co-parenting conflict over and above demographic and attachment insecurity. Taken together, these findings suggest that the scale has value in accounting for variance in co-parenting outcomes that goes beyond simply examining psychological well-being, or relationship-related affect regulation strategies. Rather, the EARDA assesses a person’s capacities contextualised to the relationship breakup. Future research using longitudinal designs will be well-positioned to examine the predictive validly of the EARDA in relation to future co-parenting outcomes.

Critiquing the scale, one might observe a predominant number of negative compared to positive items. However, this reflects the assumption that low emotional adaptation to relationship dissolution involves a complex and predominantly negative constellation of emotions. Increasing emotional adaptation involves a reduction in this negative emotional constellation, and a gradual shift towards less complex, more straightforward, beneficial, positive emotions, that by definition, require fewer items to capture.

#### Practical implications of the emotional adaptation to relationship dissolution assessment

In Study 4, we found that the upper and lower quartiles and middle half of scores on the EARDA for Sample 1 produce low, medium, and high cut-off scores that correlate highly with professional opinions of co-parenting readiness. These scores could be used by practitioners to triage clients to different levels of support. For example, parents with low emotional adaptation to relationship dissolution can be expected to have low readiness to make child-care arrangements without arguing. As clients, these parents might need support in the form of individual counselling/therapy, to help them process the negative emotions associated with the separation, and practice strategies to help reduce reactivity in communication with their ex-partner. Such interventions would have the overall goal of increasing emotional adaptation to the relationship dissolution. Parents with medium emotional adaptation to relationship dissolution can be expected to be beginning to be able to make childcare arrangements without arguing. As clients, these parents might be best supported through low intensity interventions, which might include online co-parenting programs or self-help resources. Parents with high emotional adaptation to relationship dissolution can be expected to be fully able to make childcare arrangements without arguing. As clients, these parents would need little support from practitioners, and would likely be able to cope with legally binding decision-making processes such as mediation, solicitor negotiations, or court proceedings.

#### Future research agenda for emotional adaptation to relationship dissolution assessment

We have developed the construct of emotional adaptation to relationship dissolution, and the EARDA with which to measure it. This work marks the beginning of a research agenda in which the full scope of the EARDA can be realised. One obvious next direction is to examine emotional adaptation to relationship dissolution over time. Firstly, it is important to establish how emotional adaptation changes over time, that is, the naturalistic time course of it during the separation journey. Our model proposes that generally, it increases from the time point of separation onwards, but that this increase may not be linear, and may also involve cycling back to lower levels (Fig 1). Furthermore, we proposed that individual differences in strengths and vulnerabilities would affect this rate of change. An important step in the research agenda will be to collect data to track this change, and identify the individual difference factors that affect it. Of note, these questions apply equally to non-parent and non-married participants, in whom the measure has been shown to perform just as well as in separated parents (study 3).

Secondly, an important next step in the continued validation of the EARDA will be to establish its predictive utility over time, when assessed by behavioural outcomes. To what extent does emotional adaptation at Time 1 predict behaviourally assessed co-parenting quality, or even child-related outcomes, at Time 2? Our model predicts that it does, but longitudinal data are needed to test this. Additionally, it will be important to establish whether changes in the emotional adaptation to relationship dissolution of parents over time are detectable by, and indeed affect outcomes in, those close to them, such as children. Longitudinal research will be well-positioned to address these issues.

Thirdly, future research needs to explore the potential of interventions. For example, to what extent are interventions that aim to improve co-parenting effective at increasing emotional adaptation to relationship dissolution? We propose that emotional adaptation to relationship dissolution is the mechanism by which co-parenting can be improved, hence, interventions that target emotional adaptation specifically are required in order to expedite improvements in it.

Fourthly, it is important to establish the effectiveness of the EARDA at a service level. For example, in services which might use the EARDA routinely, as part of their assessment procedures (such as Cafcass, the Children and Family Court Advisory and Support Service, a public body that represents children in family law in England and Wales) perhaps to triage clients to different levels of support, does this practice lead to improved, more efficient services, and better outcomes for individuals? An organisational approach is required to address this question.

Finally, a fundamental point for the whole future research agenda is the need to take a dyadic perspective. While it was entirely appropriate to conduct the development work of the EARDA on individuals, it is important to note that individuals do not separate in a vacuum, and there are two parties involved in a relationship breakdown. While we have identified the effects of an individual’s own emotional adaptation to relationship dissolution on their co-parenting, there may well be partner effects here too [102]. It might be that one parent having high emotional adaptation is simply not enough to lead to improvements in behavioural co-parenting outcomes. If, for example, one parent has high emotional adaptation but the other has low emotional adaptation and is consequently refusing to engage, the co-parenting efforts of one party are inherently stunted by the lack of engagement of the other parent. Furthermore, it might be that different dyadic patterns of change in emotional adaptation over time are indicative of different outcomes. For example, if one parent has a sudden increase in emotional adaptation at the same time as the other parent experiences a decline, the greater disparity between the two levels might make for increased conflict as one person attempts to work on future oriented pragmatics, and the other ruminates on events that happened prior to the breakup. Both parties could end up perceiving that the actions of the other as a personalised and vindictive blow, rather than a reflection of that individual’s emotional adaptation. Both of these scenarios could be comprehensively assessed by taking a dyadic approach to the design of the research, and using dyadic data analysis techniques, such as the Actor-Partner Interdependence Model [102].

## Conclusion

In this paper we have built on existing models of stress, coping, and relationship dissolution, to introduce the new theory and construct of emotional adaptation to relationship dissolution. We have developed and tested the Emotional Adaptation to Relationship Dissolution Assessment (EARDA), a new measure of this construct. We have also validated the measure in separated parents, and separated non-parents, and against the professional opinions of mediators in a real-world setting. Overall, our data support the assumptions of the model we have proposed. We hope that the EARDA will springboard future research into the trajectories and outcomes associates with this new construct.
